# S100B actions on glial and neuronal cells in the developing brain: an overview

**DOI:** 10.3389/fnins.2024.1425525

**Published:** 2024-07-04

**Authors:** Karina Hernández-Ortega, Arturo Alejandro Canul-Euan, Juan Mario Solis-Paredes, Héctor Borboa-Olivares, Enrique Reyes-Muñoz, Guadalupe Estrada-Gutierrez, Ignacio Camacho-Arroyo

**Affiliations:** ^1^Departamento de Biología, Facultad de Química, Universidad Nacional Autónoma de México, México City, Mexico; ^2^Department of Developmental Neurobiology, National Institute of Perinatology Isidro Espinosa de los Reyes (INPer), Mexico City, Mexico; ^3^Department of Reproductive and Perinatal Health Research, INPer, Mexico City, Mexico; ^4^Community Interventions Research Branch, INPer, Mexico City, Mexico; ^5^Research Division, INPer, Mexico City, Mexico; ^6^Department of Immunobiochemistry, INPer, Mexico City, Mexico; ^7^Unidad de Investigación en Reproducción Humana, Instituto Nacional de Perinatología-Facultad de Química, Universidad Nacional Autónoma de México, México City, Mexico

**Keywords:** S100B, astrocyte differentiation, oligodendrocyte differentiation, neurite outgrowth, neurodevelopment

## Abstract

The S100B is a member of the S100 family of “E” helix–loop- “F” helix structure (EF) hand calcium-binding proteins expressed in diverse glial, selected neuronal, and various peripheral cells, exerting differential effects. In particular, this review compiles descriptions of the detection of S100B in different brain cells localized in specific regions during the development of humans, mice, and rats. Then, it summarizes S100B’s actions on the differentiation, growth, and maturation of glial and neuronal cells in humans and rodents. Particular emphasis is placed on S100B regulation of the differentiation and maturation of astrocytes, oligodendrocytes (OL), and the stimulation of dendritic development in serotoninergic and cerebellar neurons during embryogenesis. We also summarized reports that associate morphological alterations (impaired neurite outgrowth, neuronal migration, altered radial glial cell morphology) of specific neural cell groups during neurodevelopment and functional disturbances (slower rate of weight gain, impaired spatial learning) with changes in the expression of S100B caused by different conditions and stimuli as exposure to stress, ethanol, cocaine and congenital conditions such as Down’s Syndrome. Taken together, this evidence highlights the impact of the expression and early actions of S100B in astrocytes, OL, and neurons during brain development, which is reflected in the alterations in differentiation, growth, and maturation of these cells. This allows the integration of a spatiotemporal panorama of S100B actions in glial and neuronal cells in the developing brain.

## 1 Introduction

S100B is a small Ca^2+^, binding protein belonging to a family of multifunctional proteins that includes about 25 members, with amino acid sequence homology ranging from 20 to 80%, which contain an “E” helix–loop- “F” helix structure (EF) domain that is expressed in several tissues. S100 family protein is highly conserved in vertebrate species, suggesting that it has essential conserved biological roles (Fanò et al., 1995; Gonzalez et al., 2020). S100B is the most abundant protein of this family, accounting for 96% of the S100 proteins present in the human brain and 0.5% of the total brain proteins. S100B gets its name from its solubility in a 100% saturated ammonium sulfate solution. Moore first identified it from bovine brain extract in 1965 (Moore, 1965; Isobe and Okuyama, 1978; Arrais et al., 2022). The human gene encoding S100B maps to chromosome 21q22.3 in the distal half of the long arm of human chromosome 21 (Allore et al., 1988). S100B comprises two beta subunits of 9–14 kDa, forming an acidic homodimer joined by a disulfide bond S-bond. Each monomer includes two alpha helices with a calcium-binding loop forming a conserved pentagonal arrangement around the calcium ion. This EF-hand motif is characterized by two alpha-helix structures connected by a loop with a hand-like topology. Calcium binding to EF-hand domains triggers conformational changes that allow interactions with other proteins; thus, S100B can act as calcium sensor proteins with biological activity. In addition, S100B binds zinc, suggesting that its biological actions should be regulated by this metal (Baudier et al., 2020; Singh and Ali, 2022).

S100B is concentrated in the brain tissue, particularly in astrocytes (Michetti et al., 2023). However, it is also found in oligodendrocytes (OL), Schwann cells, ependymal cells, microglia, and enteric glia (Adami et al., 2001; Vives et al., 2003). Besides, it has also been detected in low levels in particular neuronal subpopulations localized in the spinal cord, deep cerebellar, forebrain regions, and primary motor and somatosensory neocortical areas, among others (Vives et al., 2003). However, S100B expression is not restricted to nervous tissue since it is present in adipocytes, chondrocytes, melanocytes, Leydig cells, myoblasts, skeletal, dendritic cells, and some lymphocyte populations (Michetti et al., 1983, 1985, 2012; Tubaro et al., 2010).

## 2 S100B distribution in the brain

Although S100B expression is a marker of glial cells, it has also been detected in particular groups of neurons and fibers in human, mouse, and rat brains. A study focused on analyzing the distribution of S100B in different cell types of the adult human brain indicated that S100B is localized in several neural cell types and is less astrocyte-specific than glial fibrillary acidic protein (GFAP). Although cells with astrocytic morphology expressed S100B in the human cortex, 14–35% of all immunopositive cells showed oligodendrocytic morphology. However, most S100B immunostained cells resembled OL (57–97%). In addition, some of these cells showed colocalization of S100B and A2B5 (a cell surface ganglioside, marker of cells with restricted glial/neural stem cell properties), which indicates that they are O2A glial progenitors with bipotential capacity to differentiate into oligodendrocytes or type-2 astrocytes (Figarella-Branger et al., 2022). S100B was also found in ependymal cells, the choroid plexus epithelium, vascular endothelial cells, lymphocytes, and several neurons, but no microglial cells (Steiner et al., 2007).

In general, in the human brain, S100B detection has been widely reported in the hippocampus, the entorhinal cortex, and the cerebral cortex, with substantial variations between regions and cerebellum (Stagaard Janas et al., 1991b; Tiu et al., 2000, 2002; Raponi et al., 2007). More data concerning S100B distribution in rat brains is available. S100B expression at mRNA or protein levels has been reported in the midbrain, hindbrain, third ventricle, cerebellum, the pontine, facial, and motor trigeminal nuclei, and around the fourth ventricle, the forebrain, particularly, the hippocampus, neocortex, and olfactory bulb (Van Hartesveldt et al., 1986; Landry et al., 1989; Hamasaki et al., 2023; Stahlke et al., 2024). Later, in the adult rat central nervous system (CNS), two classes of S100B positive neuron populations were described, “Persistently S100B-positive” neurons, which were firmly immunoreactive and were mainly found in the nuclei of the lower brainstem and cerebellum. Moreover, “Variably S100B-positive” neurons were moderately intense immunoreactive and preferentially found in the forebrain of rats older than 90 days and were especially numerous in limbic regions (Rickmann and Wolff, 1995). In adult mouse brains, S100B expression has been reported in astrocytes and discrete neurons in the mesencephalic and motor trigeminal, facial, and lemniscus nuclei (Friend et al., 1992; Zhang et al., 2019).

## 3 S100B actions

At the intracellular level, S100B participates in various biological processes by modulating calcium signaling and interacting with diverse molecules in different cell types. In general terms, S100B is involved in cell proliferation, survival, and differentiation processes, regulates cellular calcium homeostasis and enzyme activities, and interacts with the cytoskeleton (Michetti et al., 2019).

S100B is actively released by different cell types, mainly astrocytes (Shashoua et al., 1984; Van Eldik and Zimmer, 1987; Gerlach et al., 2006; Guerra et al., 2011). S100B accumulation in the brain’s extracellular space may harm neurons, causing neuronal death (Villarreal et al., 2011). It exerts extracellular functions by binding to the multiligand Receptor for Advanced Glycation End-products (RAGE), a 45 kDa multiligand receptor’ with a transmembrane immunoglobulin-like structure. RAGE contains three extracellular (Ig)-like domains, designated V, C1, and C2, which are followed by a single transmembrane helix (TM) and a signaling domain in the short C-terminal (CT), which shares no homology with other receptor cytoplasmic domains and has no tyrosine kinase activity (Hudson et al., 2008). In turn, this CT domain recruits adaptor proteins, such as Diaphanous-1 and Toll-Interleukin 1 Receptor Domain containing Adaptor Protein (TIRAP) or the extracellular signal-regulated kinase Erk1 (Ishihara et al., 2003; Hudson et al., 2008; Sakaguchi et al., 2011). Recently, a combined approach of mass spectrometry-based methods and molecular modeling predicted the RAGE-S100B complex, suggesting S100B conformation as a tightly packed tetramer exposing a positively charged surface conformed by V domains for S100B binding. This revealed an allosteric coupling of the distal extracellular V domains to the transmembrane region, suggesting a possible mechanism for RAGE signaling across the membrane (Moysa et al., 2021). The oligomerization status of RAGE plays a major role in ligand binding and signal transduction (Wei et al., 2012; Xu et al., 2013). Once the ligand binds, RAGE initiates a complex intracellular signaling cascade, such as (ERK1/2), c-Jun N-terminal kinase (JNK), Protein kinase B (PKB/Akt) pathways, which further leads to downstream activation of transcription factors, such as nuclear factor kappa B (NF-κB), signal transducer and activator of transcription (STAT) (Jangde et al., 2020). These signaling cascades result in the production of reactive oxygen species (ROS), pro-inflammatory effects, cellular proliferation, apoptosis, and concomitant upregulation of RAGE itself (Leclerc and Heizmann, 2011; Bongarzone et al., 2017; Singh and Agrawal, 2022). RAGE activation by S100B is cell-specific and depends on the concentration of S100B (Leclerc and Heizmann, 2011). Astrocytes exposed to 50 μM of S100B mimicked several features of reactive gliosis by activating RAGE/Rac-1-Cdc42, as astroglial hypertrophy, migration facilitation, and increased mitosis. S100B also activates NF-κB, increasing RAGE proximal promoter transcriptional activity and augmenting endogenous RAGE expression (Villarreal et al., 2014). Lasič et al. (2016) have described that extracellular S100B can be re-uptaken by astrocytes by a RAGE dynamin-dependent vesicular pathway. However, it appears not to be the unique S100B receptor. In cultured myoblasts, S100B has also been shown to interact with the basic fibroblast growth factor (bFGF)/FGF receptor 1 (FGFR1) system. In particular, 1 nM S100B stimulates the mitogenic bFGF/FGFR1 signaling in low-density myoblasts (Riuzzi et al., 2011). However, continuous activation of RAGE by micromolar concentrations of S100B produces high levels of oxygen radicals, which could cause mitochondrial dysfunction and apoptosis, as its signaling pathways converge to the transcription of pro-apoptotic genes (Donato et al., 2009). Also, these S100B concentrations upregulated inducible nitric oxide synthase (iNOS), induced nitric oxide (NO) release and NO-dependent neuronal and glia death, facilitating glutamate-mediated death of neurons, upregulation of COX-II expression in microglia, increasing reactive oxygen species (ROS) production in neurons and arrest of the cell cycle (Bianchi et al., 2010). In rat astrocytes, iNOS is stimulated by S100B, which involves the transcription factor NF-kB activation signal pathway (Lam et al., 2001). In addition, oxidative stress due to ROS production induces mitochondrial DNA mutations, dysfunction of the respiratory system of mitochondria, alters membrane permeability, and affects calcium balance and mitochondria defensive system. These alterations contribute to the alterations observed in neurodegenerative diseases, such as Alzheimer’s disease (AD), Parkinson’s Disease (PD), and Amyotrophic Lateral Sclerosis (ALS) (Guo et al., 2013).

On the other hand, S100B concentrations in the nanomolar range stimulate glial cell proliferation and inhibit differentiation of rat C6 glioma cells and primary astrocytes (Selinfreund et al., 1991). They also enhance neuronal survival in chick and rat development (Winningham-Major et al., 1989; Van Eldik et al., 1991), in N18 neuroblastoma cells (Huttunen et al., 2000), and even in other cell types, such as rat L6 myoblasts (Sorci et al., 2003). Induced expression of S100B in neuronal PC12 results in enhanced proliferation and reduced differentiation and apoptosis via activation of a PI3-K/Akt/ p21WAF1/cdk4/Rb/E2F pathway (Arcuri et al., 2005). Furthermore, S100B promotes neurite outgrowth in cultures of embryonic chick cerebral cortex neurons (Winningham-Major et al., 1989) and stimulates neurite outgrowth in the rat sciatic nerve grafted with acellular muscle transplants (Haglid et al., 1997). In addition, S100B inhibits apoptosis of neurons and glial cells. In glial C6 cells, ectopic expression of S100B is associated with cell density-dependent inhibition of growth and apoptosis in response to UV irradiation (Scotto et al., 1998). Moreover, S100B treatment prevented ethanol-associated apoptosis of fetal rhombencephalic neurons (Druse et al., 2007); see more (Donato et al., 2009; Michetti et al., 2019).

S100B knockout mice (KO) compared to wild-type mice do not reveal apparent phenotype differences (Nishiyama et al., 2002b; Bluhm et al., 2015); however, mice lacking S100B showed a chronic deficiency in the blood–brain barrier (BBB), as presented by increased BBB permeability at 6 months old, which persists at 9 months old. These results suggested a key role for S100B in maintaining BBB functional integrity (Wu et al., 2016). Additionally, the results of functional studies indicate a role for S100B in modulating synaptic plasticity and hippocampus-dependent memory. S100B KO mice have higher rates of amygdala kindling and exhibit more severe seizures than their wild-type counterparts, suggesting that normal levels of S100B attenuate epileptogenesis and regulate neuronal excitability (Dyck et al., 2002). Even more, S100B acts as a modulator of neuronal synaptic plasticity; mutant mice devoid of S100B presented enhanced long-term potentiation (LTP) in the hippocampal CA1 region and had better spatial memory in the Morris water maze test and enhanced fear memory in the contextual fear conditioning (Nishiyama et al., 2002a). On the other hand, overexpression of S100B in mice led to a significant reduction in post-tetanic excitatory postsynaptic potentials (EPSP) in hippocampal slices, accompanied by substantial impairment in a spatial memory test in the Morris water maze; these changes could be due to calcium-mediated processes (Gerlai et al., 1995).

Further, the S100B protein regulates astrocyte shape and migration in human glioma cells, as reducing its levels in GL15 by Small interfering RNA technique reduces migration and acquisition of a stellate shape (Brozzi et al., 2009). It also attenuates microglia activation in GL261 murine glioma cells (Zhang et al., 2011). It was also described that S100Btg mice containing 12 copies of the murine S100B gene presented increased hippocampal progenitor cell proliferation in the subgranular zone (SGZ) and migration to the granular cell layer (GCL). Long-term exposure to S100B did not result in reactive astrogliosis (Rodrigues et al., 2022).

Besides, mice mothers immunized against S100B gave birth to neonatal mice deficient in active S100B protein, which showed adverse effects such as a slower rate of weight gain and fur coating, a deficit in visual abilities, somatosensory and posture reactions, muscular strength, locomotion, and fear/orienting processes (Davydov et al., 2015). The removal of S100B protein activity leads to slower neonatal development and the formation of a risk-tolerant (fearless) offspring phenotype. Thus, the S100B protein in infants might participate in modulating the morphological and neuromotor development, which most likely determines the formation of a behavioral phenotype (from fearless to fearful) (Davydov et al., 2015).

In perinatal medicine, some studies have associated higher levels of S100B with intrauterine growth-restricted pregnancies, reflecting chronic fetal hypoxia. Thus, the protein can be used as a biomarker of brain damage in growth-restricted newborns (Strzalko et al., 2021). Examination of S100B concentration in umbilical cord blood may be helpful to identify SGA infants at increased risk of early postnatal neurological sequelae in cases where a prenatal Doppler examination is normal, even when standard clinical and laboratory parameters are silent, and early neurological follow-up is uneventful (Gazzolo et al., 2002).

Moreover, the S100B protein is at the crossroads of several chronic pathological conditions of the nervous system; regardless of their origin, these disorders share aspects attributable to inflammation, including Alzheimer’s and Parkinson’s diseases, amyotrophic lateral sclerosis, multiple sclerosis, acute traumatic and vascular neural injury, and epilepsy. In experimental models of these diseases in general, overexpression/administration of S100B worsens the clinical presentation, while deletion/inactivation of the protein contributes to the improvement of symptoms of these diseases (Kozlowski et al., 2023; Michetti et al., 2023).

## 4 Detection of S100B during brain development

During embryonic development, the dynamic process of SNC formation requires an orchestrated interplay of biochemical and biophysical factors. S100B is found in glial cells and some neuronal populations in the nervous system. It also has significant neurotrophic and neurite outgrowth activity in developing cortical neurons and neuroblastoma cells (Kligman, 1982; Kligman and Hsieh, 1987; Winningham-Major et al., 1989). The spatiotemporal expression characterization of S100B during human brain development is not fully understood. Therefore, information derived from murine models has provided a picture of the presence of S100B in glia, in particular astrocytes and oligodendrocytes, and some neuronal groups during this critical period.

In human and rodent brain development, critical developmental processes are remarkably preserved. However, altricial rodents exhibit higher CNS immaturity at birth and accelerated postnatal development compared to humans; however, in humans, specific processes, such as neocortical myelination and synaptic maturation, extend into adulthood (Zeiss, 2021). Despite these differences, studies in rats and mice are valuable models for investigating mammalian brain development and share remarkable similarities to human brain development. A comprehensive examination of the literature pertaining to the expression of S100B in glial cells, neurons, precursors of both cell types, and fibers in the developing brains of humans, rats, and mice is presented below. Supplementary Table S1 encompassed the essential data.

Analysis of the developing human fetal brain indicated that in the hippocampus, at 6.5 weeks of gestation, radial glial cells in the tract fimbria were S100B positives (Stagaard Janas et al., 1991a). Later, the number and staining intensity of S100B positive astrocyte-like cells increased in the pyramidal layer from week 15 onwards, while the molecular and polymorphic layers maintained similar S100B immunoreactivity. The mark diminished in the molecular layer of the aged adult hippocampus, while other layers showed similar immunoreactivity to that of the late fetus. In the entorhinal cortex, at week 15, the molecular, pyramidal, and ganglionic/multiform layers showed positive S100B immunoreactivity throughout the rest of the gestation and in adult specimens. In the occipital cortex, the number of positive cells for all layers was about two-fold higher than those found in the hippocampus and entorhinal cortex, and the mark detected in the granular layer increased from week 21, reaching a plateau around week 27. S100B positive fibers were also found at week 30 but were not observed in aged adult specimens (Tiu et al., 2000).

Vinci et al. reported the earliest presence of S100B-immunopositive cells in the human frontal cerebral cortex at 11 weeks of gestation. Immunoreactivity for S100B was detected in the nuclei and cytoplasm of scattered large cells, occasionally showing an astrocytic-like appearance and mainly localized in the intermediate zone. S100B reactive cells appeared as large cells with thick cytoplasmic projections. At this age, no significant staining was observed in the ventricular zone, subventricular zone (SVZ), or cortical plate (Vinci et al., 2016). Later, at 14 weeks of gestation, the staining pattern of S100B cells in four cortical regions of the human fetal cerebral cortex showed mainly perinuclear staining, with hardly any staining in the cytoplasm. Their morphology suggests that they are immature precursors of either astrocytes or neurons. During this period, S100B-positive cells were found in the cortical plate and subcortical layers (SL), indicating a potential role in astrocyte proliferation. However, S100B-positive cells in the SL decreased after the 14th week of gestation (Tiu et al., 2002). By the 21st week of gestation, positive cells were primarily located within the cortical plate itself, with the majority being glial cells and just a few neurons. S100B positive cells progressively increased during the second trimester and peaked at 21 to 27 weeks in most cortical regions. This period is also characterized by astrocyte maturation, suggesting that S100B promotes astrocyte maturation. Significant regional differences were observed in the cortex: the motor cortex exhibited the highest counts of S100B-positive cells, while the occipital cortex had the lowest throughout gestation. This reflects differential developmental programs for different cortical regions (Tiu et al., 2002). The occipital cortex had approximately twice as positive cells for all layers as the hippocampus and the entorhinal cortex. The cortex showed no positive S100B fibers in early fetal specimens. However, these fibers became apparent at and after week 30 of gestation, which were not observed in adult specimens (Tiu et al., 2000).

Studying transgenic mice expressing enhanced green fluorescent protein (EGFP) reporter controlled by the sequence of the murine S100B gene allowed the observation of most S100B-expressing cells in the CNS. From embryonic day 13 (E13) onward, S100B was detected in neuroepithelial, glial, and neuronal cells. However, it started accumulating in CNS neurons during the perinatal period. Due to their low level and diffuse expression pattern, precise neuron mapping was possible until a few days after birth. At postnatal day 3 (P3), S100B neurons were present in the ambiguous and mesencephalic trigeminal nuclei. Neuronal EGFP/S100B expression then increased to a maximum in the fourth postnatal week (Vives et al., 2003). In adult mice, the highest level of S100B was found in ependymocytes, astrocytes, spinal cord, medullar, pontine, and deep cerebellar S100B neurons. In the forebrain, S100B was detected in neurons of the primary motor and somatosensory neocortical areas, the ventral pallidum, and the prepuberal field (Vives et al., 2003).

Regarding the distribution of S100B mRNA expression in developing rat brains, it was first detected in the germinal zone lining the fourth ventricle at E16 (Landry et al., 1989). In a later study, a very prominent S100B mRNA signal was detected earlier in E16 in a layer of cells lining the ventricle of the myelencephalon rat brain (Landry et al., 1990). In E18, S100B mRNA was noticed in the germinal zone of the third ventricle. The signal pattern and localization of S100B expression suggested a glioblast origin, specifically over Bergmann glia by P5. During the first postnatal week, a prominent induction pattern was observed in the cerebellum, the pontine, facial, and motor trigeminal nuclei, and a region surrounding the fourth ventricle. By the end of the second postnatal week, a dense punctate signal was distributed throughout the midbrain and hindbrain. S100B expression was first observed in the forebrain at E18 and corresponded to cells lining the ventricle (Landry et al., 1989). Moreover, in male rat pups, S100β gene expression and content were reported in the hippocampus, cerebellum, and cerebral cortex at P4 (Hamasaki et al., 2023). During the second postnatal week, an accumulation of S100B mRNA was observed in regions of the hippocampus, neocortex, and olfactory bulb. The distribution pattern of S100B mRNA is established during the third postnatal week and remains consistent throughout adulthood. These observations pointed to a caudal-rostral gradient in S100B mRNA expression during rat brain development, reflecting the differentiation of subpopulations of astrocytes and overall patterns of brain maturation (Landry et al., 1989).

Data obtained by immunohistochemistry for S100B allowed the visualization of a glial structure present during development within the midline raphe of the midbrain, hindbrain, and cervical spinal cord of the rat. It was composed of glial cells lying ventral to the cerebral ventricular system and for their extensive radial processes extending toward the ventral surface of the brain within the midsagittal plane. This structure was detected by immunohistochemistry from E15 (the earliest age examined) until P7–8. This glial structure was not positive for GFAP (Van Hartesveldt et al., 1986).

Together, the above data show the early presence of S100B during human (6.5 weeks of gestation), mouse (E13), and rat (E15) development. The available data on rat brain development indicate a caudal-rostral gradient in S100B mRNA expression, which reflects the differentiation course of glial subpopulations and overall patterns of brain development (Landry et al., 1989). Furthermore, the expression of S100B in these organisms is detected coincidentally in the cerebral cortex and developing hippocampus in glial and neuronal cells. This temporal coincidence suggests a possible role for S100B in the development, differentiation, and growth of these cellular subtypes, which will be discussed in the next section.

### 4.1 S100B role in glial development

Although the glioblastic origin of S100B in the developing rat brain was suggested by Landry et al. in 1989 (Landry et al., 1989), it was not until Stagaard Janas et al. (1991a,b) who described astrocytes differentiation at very early gestational ages in human fetuses that the early expression of S100B in the glial lineage was known in detail. The first glial cells appearing were vimentin-positive radial glial cells, which transit to GFAP-positive reactivity at week 8 and then transformed into astrocytes from week 14. At week 9.5, during peak neurogenesis, a population of small S100B-positive somata, distinct from GFAP-positive radial glial cells, were found in the hippocampus. These S100B positive astrocytes, at the time of their appearance, do not express GFAP or vimentin; they are thought to be derived from the ventricular zone cells and interact with the first incoming projection fibers, modulating the connectivity pattern (Stagaard Janas et al., 1991a). During development, GFAP-expressing cells in the SVZ have neural stem cell (NSC) potential; S100B is absent in these cells. Astrocytic cells in the neocortex progressively lose their neural stem cell (NSC) potential, and the onset of S100B expression characterizes a terminal maturation stage of cortical astrocytic cells. By contrast, astrocytic cells from SVZ cultured *in vitro* express S100B, as do cortical astrocytic cells, suggesting that the SVZ microenvironment represses S100B expression. Similarly, in transgenic s100b-EGFP cells, S100B expression coincides with the loss of neurosphere-forming abilities of GFAP-expressing cells. Grafting experiments indicated that S100B expression in astrocytic cells is repressed in the SVZ but not in the striatal parenchyma (Raponi et al., 2007). Transcriptomic analyses of NSCs isolated by fluorescence-activated cell sorting revealed that several astrocyte markers, including S100b and aldehyde dehydrogenase one family member L1 (ALDH1L1), were expressed in astrocytes GFAP positive but not in the SVZ NSCs (Beckervordersandforth et al., 2010).

**Figure 1:**
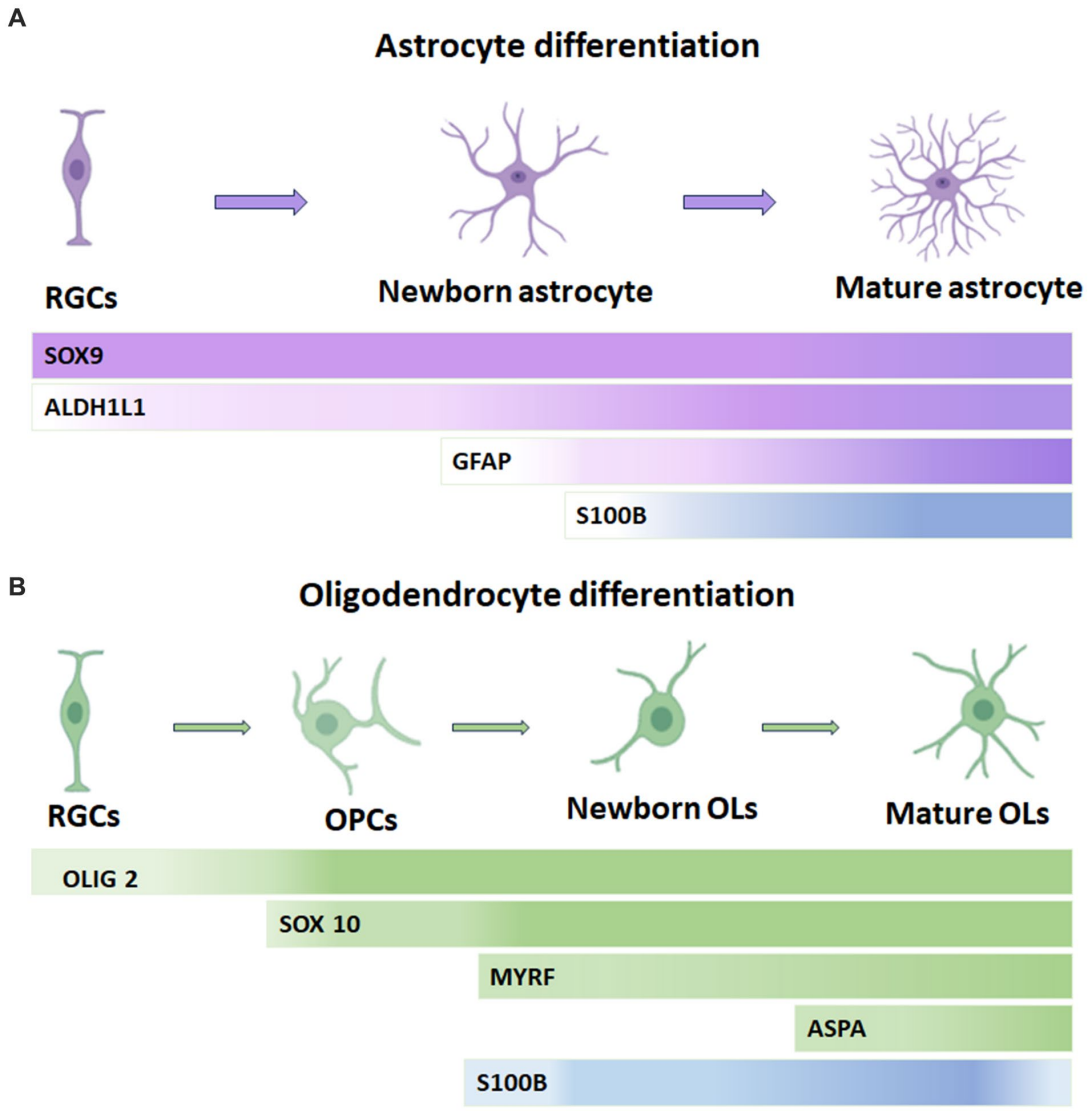
Temporary expression of S100B in oligodendrocytes and astrocytes and its correlation with other common cell lineage markers. **(A)** In the astrocytic lineage, SOX9 is considered a pan-astrocyte lineage marker and can also be found in RGCs. Similarly, ALDH1L1 is expressed throughout astrocytic development but is more abundant during differentiation into astrocytes. GFAP is present in late RGCs and reactive astrocytes. S100B expression gradually increases in astrocytes and remains sustained into adulthood. **(B)** In the oligodendrocyte lineage, OLIG2 is a pan-oligodendrocyte lineage cell that maintains its expression in OPCs but is slightly downregulated in mature oligodendrocytes. SOX 10 is a transcription factor present from the OPC stage onward. S100B expression occurs slightly earlier than MYRF and is gradually downregulated in ASPA+ mature oligodendrocytes. Ols: oligodendrocytes, OPCs: oligodendrocyte progenitor cells, RGCs: radial glial cells. ASPA: Aspartoacylase, Myelin Regulatory Factor: MYRF.

In the astrocyte lineage, S100B is gradually upregulated and maintained in adulthood. S100B is earliest expressed in the ventral neuroepithelial cells, the developing spinal cord, and later in astrocytes in the gray matter of mice. In the postnatal CNS, S100B marks nearly all protoplasmic astrocytes in the gray matter and maturing OL in the gray and white matter. Also, S100B is not expressed in radial glial progenitor cells and newborn astrocyte progenitors, as its expression in cortical gray matter astrocytes occurs only after P4 when astrocytes have already migrated from the germline region. However, the role of S100B at this neural development stage is a question that remains unknown. Figure 1A summarizes some molecular markers for astrocyte lineage cells. According to the above data and considering that S100B is expressed in the cell somata, which is suitable for cell counting, it is feasible to think that this protein remains an appropriate marker for astrocytes (Du et al., 2021; Huang et al., 2022).

Importantly, S100B expression is needed for developing adult mouse brain oligodendrogenesis. In the developing mouse brain, nuclear S100B accumulation in oligodendroglial progenitor cells (OPC) correlates with transitioning from the fast-dividing multipotent stage to the morphological differentiated, slow proliferating, pro-OL differentiation stage. In adult mice, S100B expression is down-regulated in mature OLs that have established contacts with their axonal targets, pointing to a nuclear S100B function during oligodendroglial cell maturation. The absence of S100B alters the maturation of pre-OLs *in vitro*, whereas S100B−/−mice had a delayed OPC maturation after a demyelinating insult (Deloulme et al., 2004).

Using S100B in combination with other molecular markers such as Olig2+ (a transcription factor that activates the expression of myelin-associated genes), Neural-glial antigen 2 (NG2), a membrane chondroitin sulfate proteoglycan expressed by oligodendrocyte precursor cells and 2′,3’-Cyclic nucleotide 3′-phosphodiesterase, CNP (a critical component of the molecular machinery that mediates early events in myelinogenesis), among others, has helped to discriminate OLs from astrocytes. Hachem et al. (2005) studied OL lineage development by the immunocytochemical characterization of OL-specific antigens; they described that S100B protein is expressed in postnatal and adult populations of neural precursors positive to NG2 proteoglycan both in immature and mature myelinating OLs of the brain and spinal cord of embryonic and adult mice, respectively. Later, Du et al. characterized the S100B expression pattern in the developing CNS of mice. They reported that S100B is expressed in protoplasmic astrocytes in the gray matter and myelinating or maturing OL in developing gray and white matter. S100B positive cells combined with SOX10 allow differentiating gray matter astrocytes of OLs. In addition, the time window of S100B expression in differentiating OLs and gray matter astrocytes indicates that in the former, S100B expression is slightly earlier detected than the myelin regulatory factor gene (MYRF), a transcription factor expressed explicitly by OLs at the onset of their differentiation, and gradually down-regulated in Aspartoacylase (ASPA) + mature OLs. This enzyme catalyzes the deacetylation of N-acetylaspartate (NAA) to generate acetate, which is necessary for myelination. Almost all of the S100B+ cells in the white matter are SOX10+/MYRF+ OLs from the neonatal stage to adulthood. The earliest S100B+ cells reported are differentiating OLs before P4 in the forebrain (Du et al., 2021). Figure 1B summarizes the principal molecular markers for OL lineage cells.

Furthermore, SOX10 is expressed from the early neural crest cell stage through all stages of Schwann cell development and into adulthood. The expression of S100B gradually increases during Schwann cell differentiation (Jessen and Mirsky, 2002; Gonzalez-Martinez et al., 2003). In this process, SOX10 potently transactivates the S100B promoter. Moreover, overexpressing SOX10 induces significant S100B expression in Schwann cells of dorsal root ganglia, while knocking down SOX10 with short hairpin RNA suppresses S100B expression, impairing myelination of Schwann cells. So, the SOX10-S100B signaling axis critically regulates Schwann cell proliferation and myelination (Fujiwara et al., 2014).

High levels of S100B protein in serum are presented in several perinatal inflammatory conditions involving myelin damage associated with an adverse prognosis or the appearance of sequela. Santos et al. used OL primary cultures to investigate whether excessive levels of released S100B upon early brain injury harm the neurodevelopmental period. They evaluated how elevated S100B affects oligodendrogenesis during this period. He reported that damage-induced micromolar levels of S100B impair the OL maturation course. S100B elevated concentrations decreased the transition from immature neural-glial antigen (NG2) + OPC to mature myelin essential protein (MBP) + OL and the morphological maturation of differentiated OLs. These effects were abolished by using RAGE antagonist FPS-ZM1, suggesting the participation of the S100B-RAGE axis in oligodendrogenesis impairment. Similarly, excessive S100B levels impaired oligodendrogenesis in organotypic cerebellar slice cultures, resulting in reduced myelination. Further, elevated S100B levels induced astrogliosis, NF-kB activation, and inflammation. These results point to targeting high S100B levels, and S100B-RAGE interaction may constitute efficient therapeutic strategies to reduce brain injury in the perinatal inflammatory environment (Santos et al., 2018).

### 4.2 S100B role in neuronal development

There is evidence that during CNS development, S100B might be released from glial cells and act in a paracrine way to stimulate neurite outgrowth. The first report on this matter describes a neurite extension factor (NEF) purified from soluble bovine brain extract, which promoted neurite outgrowth and rounding of the cell body in 7-day-old chick embryo cerebral cortex neurons (Kligman, 1982). Later, reversed-phase HPLC was used to purify the NEF, and an amino acid composition analysis indicated that NEF was the bovine S100B. Also, results obtained from peptide digestion indicated that neurite outgrowth-promoting activity was associated with a disulfide form of S100B (Kligman and Marshak, 1985). This observation was later expanded to neuroblastoma cells Neuro-2A, which elaborate multipolar neurites and rounded their cell body within 4–6 h of the addition of bovine S100B in a dose-dependent manner (Kligman and Hsieh, 1987). Later, it was reported that recombinant S100B, in addition to stimulating neurite outgrowth, enhanced the survival of chick embryo cerebral cortex neurons in culture (Winningham-Major et al., 1989). Overall, these results suggest potential roles for S100B in developing and maintaining neuronal function in the CNS.

Furthermore, Stagaard Janas et al. (1991a,b) analyzed the developing hippocampal fiber tract fimbria in human fetuses. After the fimbria becomes a well-defined fiber tract, a pattern demarcates the neuron-free fimbria from the hippocampus, where a mixed neuro- and gliogenesis occurs. The distinct expression of S100B in radial glial cells is restricted to the fimbria and coincidentally occurs with the establishment of hippocampal commissural connections. Thus, S100B protein *per se* seems to guide and segregate populations of growing axons of hippocampal commissural connection by providing physical and chemical cues, thereby modulating and regulating axonal growth and patterning (Stagaard Janas et al., 1991b).

Also, as previously mentioned, S100B-positive cells progressively increased in the second trimester and peaked at 21 to 27 weeks in most cortex regions (Tiu et al., 2002). Laminar formation, neurite extension, and process outgrowth occur during this period (Mrzljak et al., 1988). The second trimester is also a period of maturation for the astrocytes, indicated by the fast increase in GFAP immunoreactivity (Tiu et al., 2002; Li et al., 2021). S100B positive cells were only significantly present in the SL in week 14, suggesting that S100B probably stimulates astrocyte proliferation rather than its maturation (Tiu et al., 2002). Increased S100B during this period of neurite extension has also been reported in developing chicken embryos (Marshak, 1990). Some S100B-positive fibers at 32 weeks gestation suggest this protein’s role in fiber sprouting. In addition, S100B immunoreactivity in the frontal, motor, and temporal cortices was described as biphasic, with a peak between 21 and 27 weeks of gestation and a decline between 27 and 32 weeks. Then, a second peak occurred towards term. This S100B pattern expression inside the cortical plate resembled that of GABA. A biphasic pattern of expression could indicate different functional roles of the protein in various phases of development; in the second trimester, expression of S100B may promote neurite elongation, whereas its expression towards term may contribute to fiber sprouting and establishment of connectivity (Tiu et al., 2002).

In particular, S100B is a trophic factor essential for the normal development of serotoninergic neurons, as it increased the [3H] serotonin or 5-hydroxytryptamine (5-HT) uptake capacity by the cultured serotoninergic neurons and significantly increased (135 and 147%) the neurite length 30 h after S100B application (Azmitia et al., 1990). Also, S100B increased considerably neurite length, the number of primary neurites, and the number of terminal neurite segments (neurite complexity) in 5-HT neurons from E14 of the rostral raphe. As S100B immunoreactivity is observed in glial cultures derived from rostral raphe, S100B may have autocrine effects on glia and paracrine actions on serotoninergic developing neurons (Liu and Lauder, 1992). During the development of the 5-HT system, a substantial number of S100B-positive cells is found in the midline raphe glial structure (MRGS), a sizeable transient structure in the brainstem that spans multiple serotoninergic nuclei, including the dorsal raphe, median raphe, and B9 complex. During the critical period in developing serotoninergic neurons, S100B immunoreactivity in the MRGS is detected from E15 and disappears from P5 to P8. In addition to providing trophic support for developing 5-HT neurons, this structure could guide neuronal migration and exert a function similar to radial glia (Van Hartesveldt et al., 1986). MRGS also has a high concentration of S100B in the ependyma of the third and fourth ventricles and the aqueduct. S100B immunopositive ependymal cells may influence the development of the serotoninergic neurons in the supra- and subependymal plexuses, which innervate ventricles since the guidance of axonal growth cones is facilitated by proteins secreted by the ependymal cells (Sarnat, 1998).

Moreover, it was reported that the homozygote mouse strain with genetic polydactyly, Polydactyly Nagoya (Pdn), shows a significant decrease of S100B positive cells and in the development of serotoninergic fibers in the hippocampus and caudo-dorsal cortex at P1 (Ueda et al., 1994). By contrast, Nishiyama et al. (2002b) described that the distribution of serotoninergic fibers, determined by immunohistochemistry in the brains of S100B KO at P7 and P60, was quite similar to that of wild-type mice; these findings argue against the postulate that S100B has an essential role in neurite development of serotoninergic neurons *in vivo*. The Brain-derived neurotrophic factor (BDNF), like S100B, has a trophic effect on serotoninergic neurons (Nishi et al., 2000). Heterozygous mice (BDNF1/2) exhibit decreased serotoninergic innervation in aged (18-month-old) animals (Ernest Lyons et al., 1999). S100B and BDNF could exert complementary maturation effects on cultured serotoninergic neurons (Nishi et al., 2000). Therefore, BDNF may compensate for the lack of s100B in the KO mouse for this protein.

Besides, it is known that granule cell proliferation, migration, and dendritic growth of Purkinje cells occur at P5 and P10 in the cerebellum. In addition, Bergmann glial cells contain an exceptionally high concentration of S100B and serve as a scaffold for migrating granule cells and growing Purkinje cell dendrites. However, S100B deficient mice have normal cerebellum development, and knockout mice performed equally in locomotor behavior tests (Bluhm et al., 2015).

Although, so far, we have information about the promoting actions of S100B on the development and activation of astrocytes, OLs, and the stimulation of dendritic development during embryonic development, the exhaustive study of temporal and spatial S100B activity is necessary to obtain a comprehensive view of the divergent roles of this protein in the differentiation process of neurons and glial cells, and the precise timing of S100B actions along neurodevelopment.

## 5 Modifications in S100B expression are associated with neurodevelopmental alterations

**Figure 2:**
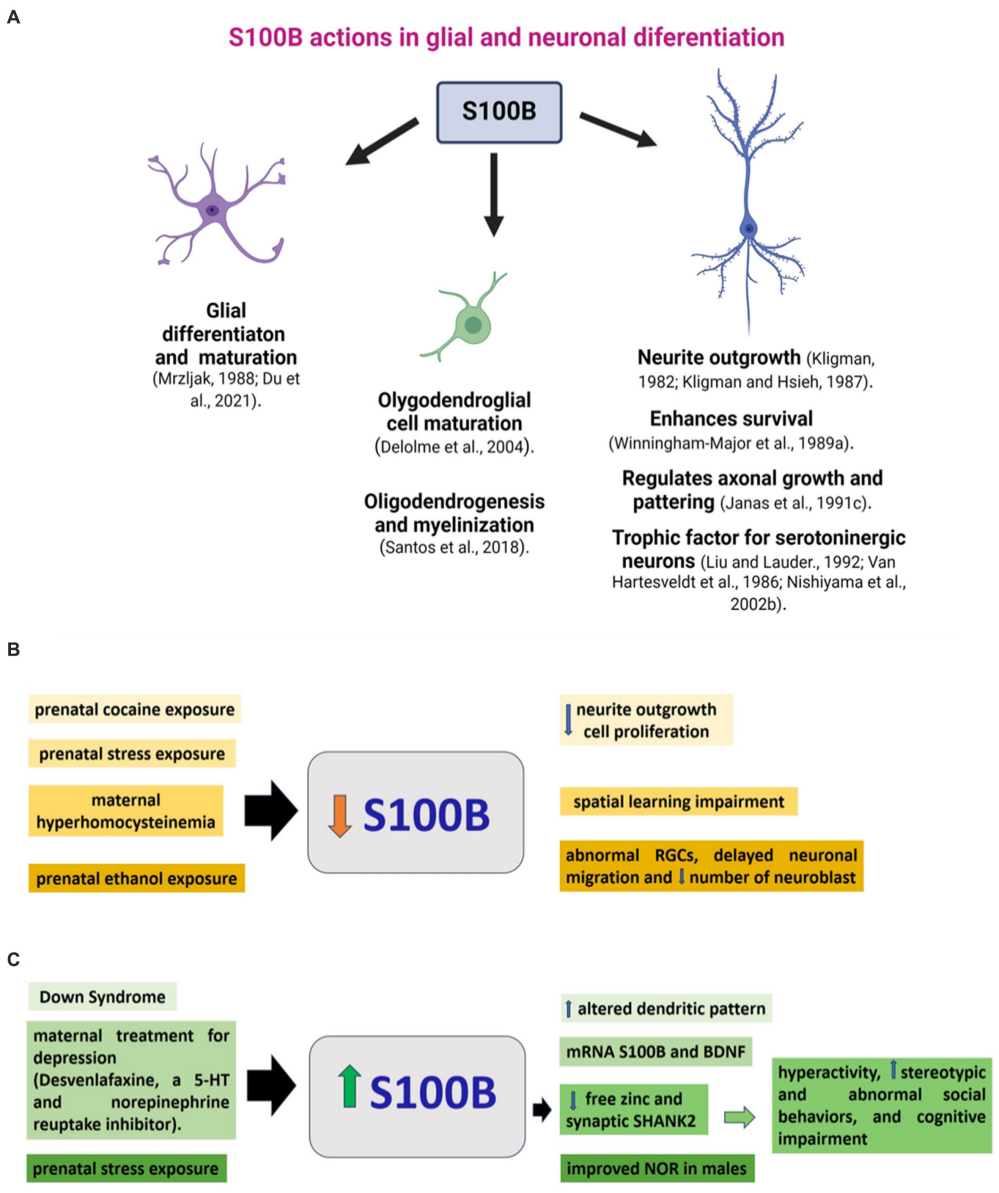
S100B during brain development. **(A)** S100B actions on glial and neuronal development described in animal models. Changes in S100B are related to molecular, cellular, or functional alterations in brain development. **(B)** Conditions such as prenatal or neonatal drug exposure or maternal pathology that cause a decrease in S100B levels in the brain, along with corresponding neurodevelopmental changes, are organized and presented in similar colors. **(C)** Elevated levels of s100B in the brain, commonly linked to genetic conditions or prenatal exposure, correlate with molecular, cellular, or functional alterations in neurodevelopment. Stimuli and their respective impacts are displayed in matching colors. Further information on the reports above can be found in the main text. PCPA: parachlorophenylalanine, RGCs: radial glial cells, NOR: novel object recognition.

S100B participates in glial differentiation and has a trophic role during CNS development, which includes neuronal survival, regeneration, and promotion of neurite extension (Michetti et al., 2012; Gazzolo et al., 2019). It also plays a role in lesion-induced collateral sprouting and reactive synaptogenesis (McAdory et al., 1998). By contrast, its overexpression can have dangerous effects, activating iNOS and the subsequent production of NO with astrocyte cell death (Lam et al., 2001). As S100B is essential in normal brain development, alterations in its expression and function affect neurodevelopment. Next, significant examples of alterations in S100B in the brain associated with neurodevelopmental changes in mammalian models and humans are reviewed (Figure 2).

Prenatal cocaine administration in rats decreased cell proliferation, delayed neurite outgrowth, increased density of vimentin-positive radial glial cells, and diminished S100B immunoreactivity in 6-day-old male offspring. Reduced S100B presence would contribute to delayed neurite outgrowth and decreased glial proliferation caused by prenatal exposure to cocaine. Given the pivotal role these glial cells play in neuronal development as guidance fibers and a source of trophic factors, any delay in their development could have profound implications for overall brain development (Clarke et al., 1996) (Figure 2A).

The S100B gene is located in the 21q22 region, a chromosomal locus whose duplication has been associated with the occurrence of Down Syndrome (DS). This trisomic state has a consequent high S100B expression (Allore et al., 1988). Consistently, DS astroglia expressed a much higher level of S100B than astroglia in control brains. Although the immunocytochemical distribution of S100B in neural cell types in DS does not differ from that in control brains (Michetti et al., 1990), its expression at mRNA and protein levels and the number of S100B-immunoreactive astrocytes are markedly higher in DS brains (Jrgensen et al., 1990; Goodison et al., 1993; Chen et al., 2014). In DS fetuses, neonates, infants, and adults, S100B immunoreactive cells at the temporal lobe were at least twice that in non-DS. In postnatal DS, intensely immunoreactive cells corresponded to reactive astrocytes (Griffin et al., 1989). In the same way, a two-fold number of S100B-positive cells was observed in the hippocampus, frontal lobe, and calcarine cortex of DS compared to in fetus and adult controls (Mito and Becker, 1993). In light of the altered dendritic pattern in DS, this could be related to the high levels of S100B, which has a critical role in neuritic growth (Figure 2B). Moreover, DS astroglia expresses higher levels of S100B, GFAP, and ROS, indicating that DS astroglia is in a reactive state. Inhibiting the overexpression of S100B with a small interfering RNA in DS astroglia reversed its pathological phenotype (Chen et al., 2014).

Prenatal stress disturbs neonatal rat brain development. During the last week of pregnancy, pregnant female rats were individually restrained three times a day (45 min) and simultaneously exposed to bright light, which reduced 25% of rat hippocampal S100B content (Figure 2A). Further, male hippocampal S100B content was negatively correlated with plasma corticosterone levels. Interestingly, positive correlations were found between female S100B levels, fetal growth, and hippocampal BDNF content. As a consequence of prenatal stress, the reduction in neonatal hippocampal S100B levels may affect postnatal brain development (Van Den Hove et al., 2006). Also, prenatal stress in rats did not affect novel object recognition in females but improved it in males, and led to an increase in Doublecortin+, the number of astrocytes GFAP+, and the intensity of GFAP and S100B immunofluorescence in the dentate gyrus (Barbie-Shoshani et al., 2016). These data suggest a sexual dimorphism in the effects of prenatal stress and the complex action in S100B on neural development. Learning deficits are accompanied by significant reductions in the expression of GFAP and S100B in the brains of maternally hyperhomocysteinemic pups on P1 and an altered expression pattern of neural cell adhesion molecule (NCAM) (Baydas et al., 2007).

Pre- and postnatal protein malnutrition in rats increased GFAP and S100B expression in the cerebral cortex, hippocampus, and cerebellum at birth, suggesting the presence of astrogliosis. However, no changes in the content of GFAP and S100B were found on the P60 in malnourished rats (Feoli et al., 2008). Prenatal ethanol exposure induces morphological and functional alterations in CNS development. One month before mating and along pregnancy and lactation, female Wistar rats were fed 5.9% (w/w) ethanol, resulting in moderate blood EtOH concentrations. In the cerebral cortex of E12-P3 rats, radial glial cells presented a reduction in vimentin, nestin, S100B, and Pax6 levels and had abnormal morphology. In addition, they showed a marked delay in neuronal migration, decreased numbers of neuroblasts, and Pax6+ cells in the cerebral cortex. These observations indicate the occurrence of a sequence of toxic events that may contribute to the development of cortical dysplasia in offspring exposed to ethanol during gestation (Aronne et al., 2011).

Depression during pregnancy has adverse effects on fetal development. Prenatal desvenlafaxine, a serotonin and norepinephrine reuptake inhibitor used to treat gestational depression (from E5-E20), induced upregulation of mRNA of BDNF and S100B expression in the fetal rat brain; such upregulation in the gene expression depended on the dose of the drug and may lead to a delay of brain development (Ahmed et al., 2022) (Figure 2B).

As previously mentioned, 5-HT is a regulator of the development of neurons, and this function is associated with the release of S100B from astrocytes through actions of the 5-HT1A receptor (Whitaker-Azmitia et al., 1990). S100B acts as a trophic factor on 5-HT neurons (Azmitia et al., 1990) and stimulates neurite outgrowth (Kligman and Marshak, 1985) and the refinement of cortical circuitries (Müller et al., 1993). *In utero*, ethanol exposure significantly reduced S100B immunopositive glial cells in the MRGS at E20 and the dorsal raphe at P2. In addition, the treatment of pregnant rats with Ipsapirone, a 5-HT(1A) agonist between E13 and E20, prevented the ethanol-associated reduction of S100B immunopositive glial cells. Thus, a decrease of S100B mediates some of ethanol’s damaging effects on developing 5-HT neurons, and some of the protective effects of maternal 5-HT(1A) agonist treatment are related to increasing S100B glial levels (Eriksen et al., 2000).

In this context, it is relevant to indicate that copy-number variations of S100B are related to the etiology of autism spectrum disorders (ASD), a group of neurological disorders associated with synaptic dysfunction (Egger et al., 2014). S100B is a calcium and zinc binding protein (Hagmeyer et al., 2018) that may have neuroprotective actions since acutely elevated levels of S100B buffer zinc and regulate calcium-signaling, which eventually diminishes excitotoxicity (Hagmeyer et al., 2018). In addition to acting as a neuromodulator, zinc has a role in neuronal differentiation, synapse formation, and plasticity (Skalny et al., 2021). In particular, synaptogenesis and function glutamatergic terminals are affected by zinc signaling that also regulates the dynamics of SHANK2 and SHANK3, scaffold proteins at the postsynaptic density (Grabrucker et al., 2011; Grabrucker, 2014). SHANK2 and SHANK3 are significant autism candidate genes (Leblond et al., 2014; Kreutzmann et al., 2024) whose levels are affected by S100B in a zinc-dependent manner. Daini et al. reported that mice exposed to high S100B levels in late embryonic development exhibit low levels of free zinc and SHANK2 in the brain. These animals display hyperactivity, stereotypic and abnormal social behaviors, and cognitive impairment (Daini et al., 2021). Thus, when S100B levels increase in pro-inflammatory conditions, there is a decrease in zinc levels and, therefore, a reduction of SHANK2, related to synaptopathies in ASD (Figure 2B). Thus, S100B may be an essential drug target for ASD treatment strategies (Daini et al., 2021). The variety of alterations in the neural development of offspring with adverse prenatal or genetic conditions described above and their relationship to changes in S100B levels demonstrates the diversity of actions of this protein on glial and neural development, which is not yet fully understood.

## 6 Concluding remarks

The significance of S100B actions during brain development is notably demonstrated in its crucial function in OL differentiation. Besides, the importance of S100B’s expression during the astrocytic maturation stage prompts inquiries into its role in this developmental stage of the astrocytic lineage. When considering the functions of S100B in neuronal differentiation, it is advisable to identify the specific periods during brain development when S100 activity is crucial. While S100B’s primary actions pertain to axonal growth, a clear definition of its fundamental role in brain structure formation is necessary. In this context, the comprehensive depiction of the mouse model demonstrating alterations in the development of SHANK protein-positive synapses due to high levels of S100B during pregnancy, altering calcium homeostasis, and the consequent behavioral changes associated with ASD hold significant value.

This current scenario raises the need for further detailed studies, such as the one mentioned above. These studies are necessary to establish the broad signaling cascades of S100B actions and their timing in regulating critical aspects of glial and neuronal differentiation, which can be affected by adverse genetic or prenatal conditions.

Taken together, this evidence relates the key role of the expression and early actions of S100B in astrocytes, OLs, and certain neuronal groups during brain development, which are reflected in the alterations observed in differentiation, growth, and maturation of these cells caused by changes in the expression of this protein. In addition, it raises new questions about the actions of S100B in specific regions and periods of brain development, allowing the integration of a spatiotemporal panorama of the actions of S100B in glial and neuronal cells in the developing brain. In the future, S100B actions will allow the identification of potential therapeutic targets for impaired neurodevelopmental conditions.

## Author contributions

KH-O: Writing – review & editing, Writing – original draft, Visualization, Investigation, Conceptualization. AC-E: Writing – review & editing, Investigation. JS-P: Writing – review & editing. HB-O: Writing – review & editing. ER-M: Writing – review & editing. GE-G: Writing – review & editing. IC-A: Writing – review & editing, Supervision, Project administration, Funding acquisition.
