# Supplementary material for: S100B actions on glial and neuronal cells in the developing brain: an overview
Source: Front Neurosci. 2024 Jul 4;18:1425525. doi: 10.3389/fnins.2024.1425525 (PMC11256909; doi:10.3389/fnins.2024.1425525)
Supplement: Supplementary file 1 [file Table_1.DOCX]

Supplementary Material

# Supplementary Table 1

|  | **Presence of S100B during brain development** | | | | |  |  |
| --- | --- | --- | --- | --- | --- | --- | --- |
|  | **Neuron** | **Glia** | **Others** | **Age** | **Reference * Notes** |  |  |
| **Human brain** |  | Radial glial cells in hippocampal fiber tract fimbria. |  | At week 6.5. | ***Janas et al., 1991.*** |  |  |
|  |  | Astrocyte cells in the pyramidal, the molecular and polymorphic layer of the hippocampus. |  | From week 15 onwards and aging. | ***Tiu et al., 2000.*** |  |  |
|  | Positive S100B cells in the entorhinal cortex's molecular, pyramidal, and ganglionic/multiform layers. | |  |  |  |  |  |
|  | Positive cells in all layers in the occipital cortex. | |  | From week 15, it increased at week 21 and reached a plateau at week 27. |  |  |  |
|  |  |  | S100B positive fibers. | At week 30. |  |  |  |
|  | 1. Immature precursors of either astrocytes or neurons in cortical plate and the Subcortical layers. 2. Glial cells and a few neurons inside the cortical plate. | |  | 1. At week 14. 2. Peaks weeks 21-27. | ***Tiu et al., 2002.*** |  |  |
|  |  | | S100B^+^ fibers in the frontal, motor, and temporal cortices. | First peak weeks 21-27. The second peak occurred towards term. |  |  |  |
|  | Immunoreactive S100B cells in the intermediate zone (corticogenesis). | |  | 11 weeks of gestation | ***Vinci et al., 2012*** |  |  |
| **Rat brain** |  | Glial cells in the midline raphe of the midbrain, hindbrain, and cervical spinal cord. |  | P5 | ***Van Hartesveldt et al., 1986.*** |  |  |
|  |  | Germinal zone lining the fourth ventricle. |  | E16 | ***Landry et al., 1989 ****S100B mRNA |  |  |
|  |  | Germinal zone of the third ventricle. |  | E18 |  |  |  |
|  |  | Bergmann glia |  | P5 |  |  |  |
|  | 1. Cerebellum, the pontine, facial, motor trigeminal nuclei, and the fourth ventricle. 2. Midbrain and hindbrain 3. Cells lining the ventricle. 4. Hippocampus, neocortex, and olfactory bulb. | |  | 1. P1 week. 2. P2 week. 3. From E18 until P2 week. 4. End of P2 week. |  |  |  |
| **Mouse brain** | Neuroepithelial, glial, and neuronal cells with diffuse expression pattern | | | E13 | ***Vives et al., 2003****. **Transgenic mice expressing EGFP controlled by the S100B gene |  |  |
|  | Neurons from the ambiguous nucleus and the mesencephalic trigeminal nucleus. |  |  | From P3 to P4 week. |  |  |  |
|  |  | 1. Oligodendroglial precursors on the midline in the ventral medulla. | 2. Brain stem white matter tracts (fimbria and corpus callosum). | 1. E13. 2. P3. |  |  |  |
|  | **Supplementary Table 1**. This table summarizes reports on the presence of S100B at the protein or mRNA level during human, rat, and mouse brain development. If available, the cell type, site of finding, age, and literature reference are described in each case. | | | | |  |  |
|  |  |  |  |  |  |  |  |

**
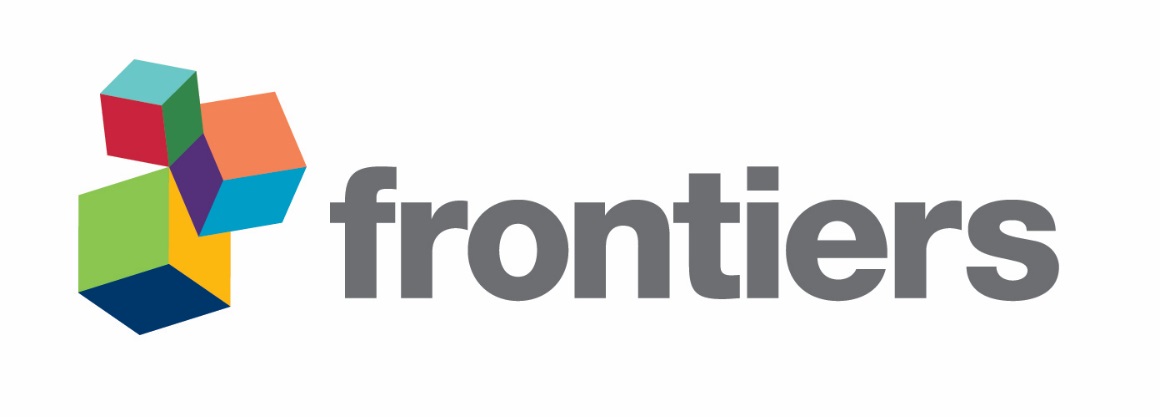
**
